# Touching Soma Segmentation Based on the Rayburst Sampling Algorithm

**DOI:** 10.1007/s12021-017-9336-y

**Published:** 2017-09-22

**Authors:** Tianyu Hu, Qiufeng Xu, Wei Lv, Qian Liu

**Affiliations:** 1 Britton Chance Center for Biomedical Photonics, School of Engineering Sciences, Wuhan National Laboratory for Optoelectronics-Huazhong University of Science and Technology, Wuhan, 430074 China; 2 MoE Key Laboratory for Biomedical Photonics, Huazhong University of Science and Technology, Wuhan, 430074 China

**Keywords:** Image analysis, Soma segmentation, Rayburst sampling algorithm, Distance transform

## Abstract

Neuronal soma segmentation is essential for morphology quantification analysis. Rapid advances in light microscope imaging techniques have generated such massive amounts of data that time-consuming manual methods cannot meet requirements for high throughput. However, touching soma segmentation is still a challenge for automatic segmentation methods. In this paper, we propose a soma segmentation method that combines the Rayburst sampling algorithm and ellipsoid fitting. The improved Rayburst sampling algorithm is used to detect the soma surface; the ellipsoid fitting method then refines jagged sampled soma surface to generate smooth ellipsoidal shapes for efficient analysis. In experiments, we validated the proposed method by applying it to datasets from the fluorescence micro-optical sectioning tomography (fMOST) system. The results indicate that the proposed method is comparable to the manual segmented gold standard with accurate soma segmentation at a relatively high speed. The proposed method can be extended to large-scale image stacks in the future.

**Electronic supplementary material:**

The online version of this article (10.1007/s12021-017-9336-y) contains supplementary material, which is available to authorized users.

## Introduction

Neuronal morphology quantification analysis plays an important role in neuroscience, such as neuron classification, dynamic analysis, electrophysiology simulation, and even understanding the relationship between functions and structures in the brain (Chen et al. 2012; Svoboda 2011; Yan et al. 2013; Ascoli et al. 2001; Sholl 1953). Neuronal soma morphology characteristics such as the soma location and size are important indices for neuron morphology quantification (Meijering 2010).

In recent decades, rapid advances in optical imaging technology have generated large amounts of data for neuron morphology research (Peng et al. 2015; Peng and Long 2010; Peng et al. 2010). This has made manual analysis methods too time-consuming to achieve a high throughput despite being the best way to get accurate results (Saraswat and Arya 2014). Consequently, much effort has been focused on developing automatic soma reconstruction methods. Many efficient algorithms have been proposed, such as the watershed transform (Lin et al. 2003), graph cut-based method (Alkofahi et al. 2010), and clustering-based method (Liu et al. 2008). However, many grayscale-based algorithms were designed for two-dimensional image data, and quite a few of them can be directly extended to three dimensions because of the intensity anisotropy in light microscopy imaging data (He et al. 2014). Low-quality images and clustered somata are further challenges (Saraswat and Arya 2014). Therefore, an efficient segmentation method would require a combination of multiple methods.

Cell segmentation methods developed in recent years have combined many algorithms (Meijering 2012). For example, methods that use different detection algorithms for isolate and touching cells have exhibited very good performance (Xu et al. 2014). Guo et al. (2014) proposed a method that uses a Bayesian network and the watershed algorithm to separately treat isolated and touching cells. Alkofahi et al. (2010) proposed a semiautomatic method that combines the initial segmentation algorithm for seed point detection and the graph-cut algorithm for boundary segmentation. He et al. (2014) proposed a method using the concave point clustering method for detecting touching somata and using the random walk method for cell segmentation. However, efficient segmentation of both isolated and touching somata could still be a problem for large light microscopy imaging datasets.

The Rayburst sampling algorithm is a rapid method for convex structure detection (Wearne et al. 2005; Rodriguez et al. 2006). In our previous work, the Rayburst sampling algorithm was used for neurite tracing and proved to be highly accurate (Ming et al. 2013). However, this version of the Rayburst algorithm is not suitable for neuron soma segmentation. Yan et al. (2013) proposed an improved Rayburst sampling algorithm for soma segmentation of a Golgi stained dataset that demonstrated high performance for hollow somata. However, this method cannot process closely touching somata well. The main problem is that the rays always stop at the wrong position by the intensity threshold (Quan et al. 2014).

In this paper, we propose a method for neuronal soma segmentation from light microscope images. The proposed method combines an improved Rayburst sampling algorithm and ellipsoid fitting method. This improved Rayburst sampling algorithm can detect the surface of touching somata from soma centroids detected by the distance transform based method, and the ellipsoid fitting method is used to generate smooth segmentation results based on sampling results from the Rayburst sampling algorithm. This method was validated by using datasets from the green fluorescence micro-optical sectioning tomography (fMOST) system (Gong et al. 2013) and volume-object annotation (VANO system (Peng et al. 2009).

## Method

### Method Overview

**Fig. 1:**
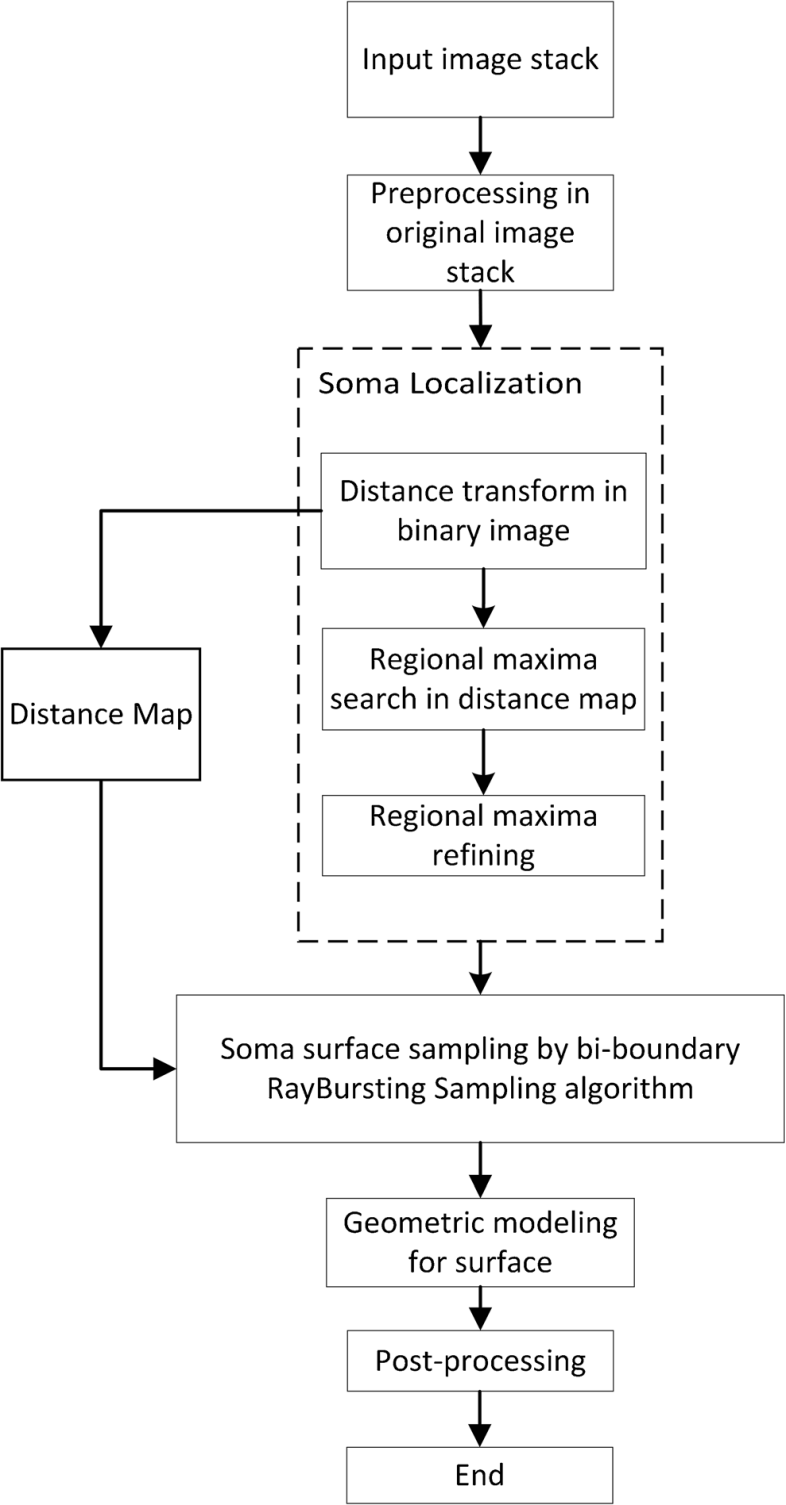
Flowchart of the proposed method

The flowchart of the proposed method is shown in Fig. 1. In the image preprocessing step, the image stack is enhanced by multi-scale Laplace of Gaussian (LoG) filters. Then, an adaptive thresholding method (Otsu 1979) used to extract the soma region. The soma localization step includes the Euclidean distance transform (EDT) and regional maxima search. These regional maxima in the distance map are identified as soma location candidates by using the H-dome transform based on EDT. Finally, soma segmentation is performed with the improved Rayburst sampling algorithm followed by the ellipsoid fitting method.

### Image Preprocessing

The resolution of the original image stack from the fMOST dataset was anisotropic (0.5 μm × 0.5 μm × 2.0 μm). We converted the resolution to isotropic (0.5 μm × 0.5 μm × 0.5 μm) by bilinear interpolation of slices of the fMOST dataset. The resolution of the VANO dataset was isotropic (0.24 μm × 0.24 μm × 0.24 μm) and thus did not need to be converted.

In this study, the image foreground regions contained somata, and the background contained unrelated structures. In the fMOST data, the somata and several neurites generally had a relatively high intensity. We used a multi-scale LoG filter to enhance the soma regions.

The improved filter is defined as

1 $$ {I}_{bg}=I-{\sum}_{i=1}^k{LoG_{\sigma (i)}}^{\ast }I $$

2 $$ {I}_{fg}=I-{I}_{bg- positive} $$

where *I* is the original image, *I*_*fg*_ is the foreground, *I*_*bg*_ is the background, and *LoG*_*σ*(*i*)_ denotes the operator of the LoG filter with the sigma value *σ*(*i*). *I*_*bg* − *positive*_ is the positive elements of *I*_*bg*_, and the negative elements are set to zero. *k* is the subscript of the maximum filter scale *σ*(*k*).

The sigma value *σ* is set based on the soma radius, and LoG filters with different *σ* may enhance structures with different sizes. Subtracting the filtered image from the original image can weaken the image background.

This makes the image foreground clearer than before, and an adaptive thresholding method (Otsu 1979) can be used to extract the image foreground. Finally, holes in somata are filled, and small regions (less than 200 voxels) are deleted to refine the foreground.

### Soma Localization

**Fig. 2:**
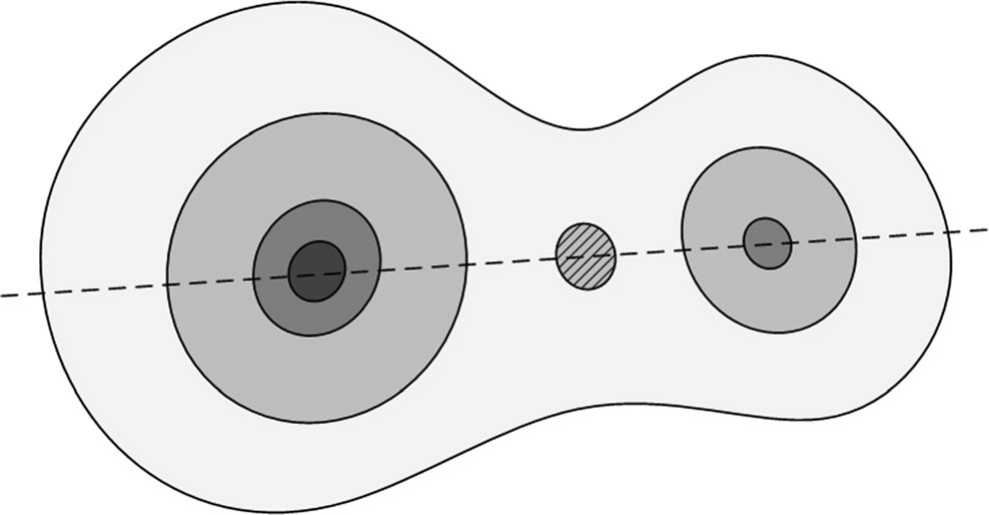
Jitter in distance map: Two somata on a distance map. The light intensity indicates a small distance value. The region between the two soma centroids is the jitter

Soma locations are regarded as the starting points for segmentation. Generally, soma locations can be described as soma centroids; these points always have a long distance to the background. Distance transform (DT) can be used to evaluate the shortest distance value from foreground voxels to background voxels. The regional maxima in the distance map are candidates for soma centroids. The H-dome transform (Vincent 1993) is used to eliminate reluctant regional maxima (regarded as jitter, as shown in Fig. 2) and provide candidate soma locations for surface detection.

### Soma Surface Detection

The improved Rayburst sampling algorithm is based on DT instead of image intensity. The somata in the image stack from the fMOST dataset were solid with similar intensities, so the boundaries between touching somata were always unclear.

**Fig. 3:**
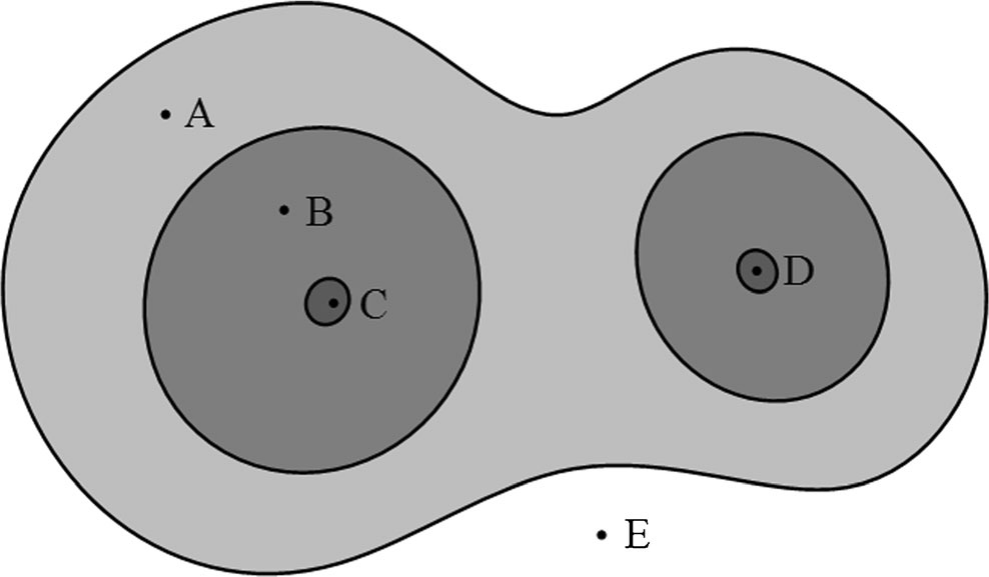
Four types of points on a distance map. A lighter intensity represents a smaller distance value

**Fig. 4:**
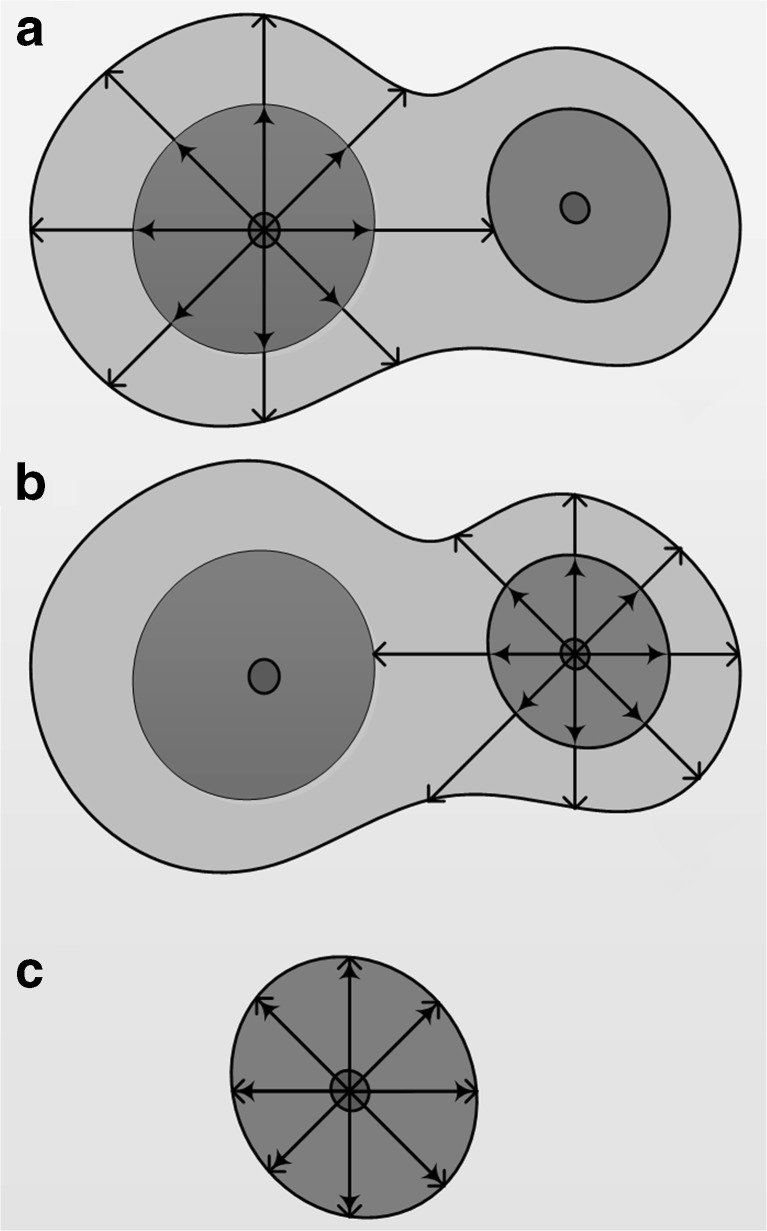
Soma surface sampling for (a, b) touching somata and (c) isolated somata

In a distance map, a distance value is defined as the shortest distance from the foreground voxels to the background region. Voxels near soma centroids can have a larger distance value than voxels near the foreground boundary. Under the assumption that a soma is shaped like an ellipsoid, the touching region is concave and narrow, as shown in Fig. 3. Voxels in this region are closer to the background than inside voxels, so the real boundary between touching somata can be around the regional minima of the distance map in the touching region, as shown in Figs. 4(a) and (b). These regional minima can serve as good stopping positions for rays.

For convenient analysis, we utilized the idea of a basin and simulated rainfall in a watershed transform. As shown in Fig. 3, foreground voxels can be classified into four types based on different locations in the foreground. We assumed that simulated rainfall flows along the opposite direction of the distance gradient and that the rain can eventually reach the regional maxima of the image.

The four types of voxels in the foreground are as follows. (1) Type 1 is regional maxima, which include soma centroids and are regarded as the starting points for sampling rays; see regions C and D in Fig. 3. (2) Type 2 is voxels around the soma centroid that do not stretch to other touching somata to form the distinct region of one soma; see region B in Fig. 3. (3) Type 3 is voxels of region A between the boundary region B and background. This region stretches from one soma to the other of a touching soma pair. This region is closer to the background than type 2. It contains the touching parts of two somata as well as uncertainty; see region A in Fig. 3. (4) Type 4 is background voxels; see region E in Fig. 3.

The soma centroids detected during the soma localization step are regarded as type 1 voxels, and the sampling ray starts from these voxels. As shown in Fig. 4, the difference between the touching and isolated somata is type 3 voxels. Touching somata contain uncertain regions with type 3 voxels. The best stopping positions for rays can be set at regional minima in type 3 voxels for touching somata and type 2 voxels for isolated somata.

For this reason, we defined two kinds of boundaries for sampling rays to detect the soma surface. The inner boundary is between the type 2 and 3 regions for one soma; see the inside arrows in Fig. 4(a). The outer boundary is between a type 3 region and background; see the outside arrows in Fig. 4(a). As shown in Figs. 4(a)–(c), the outer boundary reflects the final contour for isolated and touching somata. The outer and inner boundaries especially coincide with each other for an isolated soma. As noted earlier, the outer boundary can be set to the regional minimum at the touching part or background for touching somata.

Overall, rays run along the direction of the sampling core until the distance value increases or becomes zero. The stopping conditions for sampling rays can be set as follows: (1) the rays reach the background voxels while the distance value is zero, and (2) rays reach the regional minima in the touching region while the distance value increases.

**Fig. 5:**
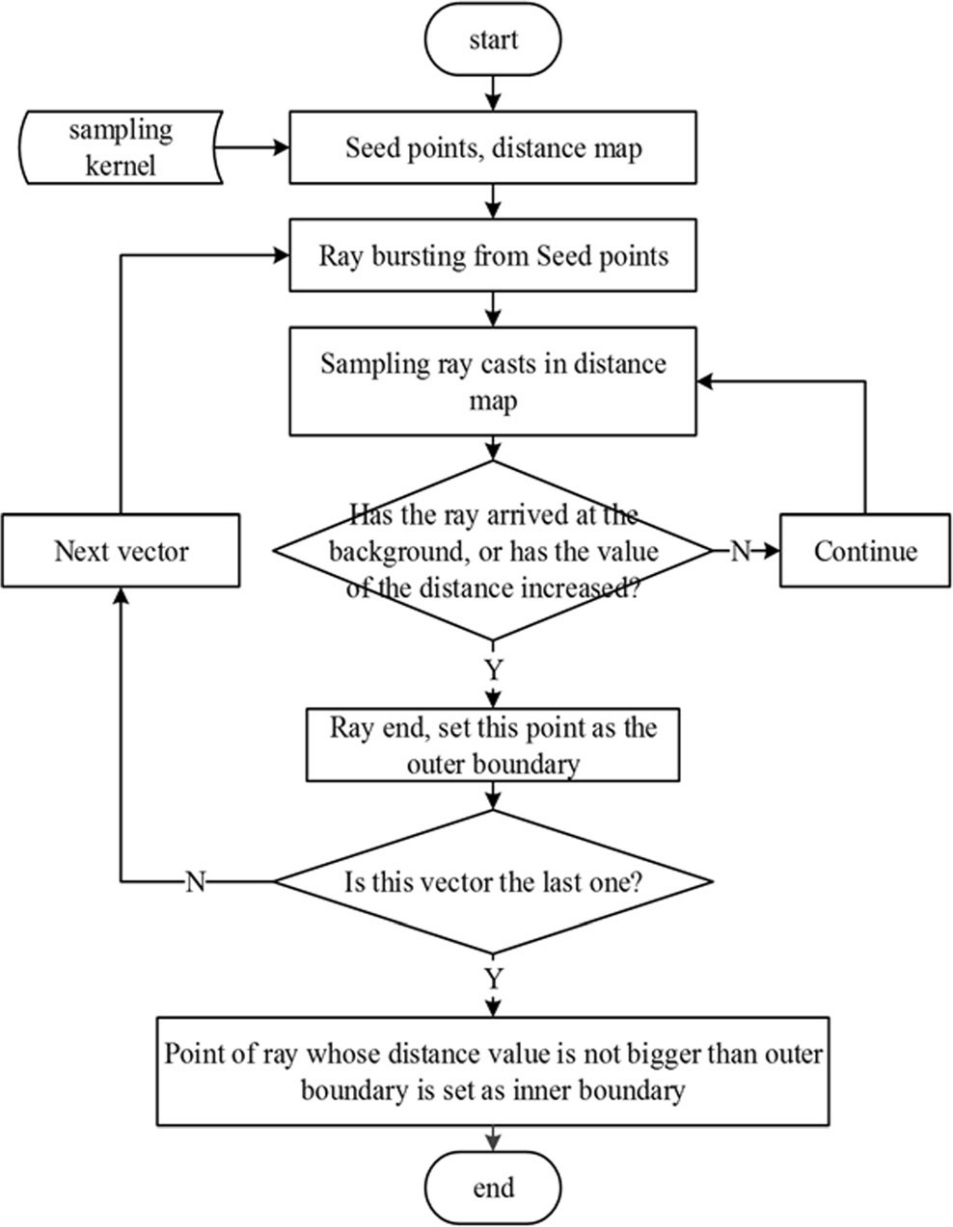
Flowchart of the improved Rayburst sampling algorithm

In contrast, the inner boundary can be set at a position where the distance value does not change, or it would coincide with outer boundary. This boundary could be used to fix the outer boundary. The complete flowchart for this algorithm is shown in Fig. 5.

Finally, the actual sampled boundary consists of discrete voxels around the soma surface. As described in the next section, we use an ellipsoid fitting method to generate the segmentation result and a volume threshold to filter the results.

### Soma Shape Fitting

Most somata in the fMOST data are shaped like ellipsoids, so we propose an ellipsoid fitting method to generate soma segmentation results based on the sampling results from the method described in the previous section.

The B2AC method for the direct least squares fitting of ellipses was proposed by Fitzgibbon and is widely used for two-dimensional image data. Li and Griffiths (2004) discussed the constraint conditions for ellipsoids and extended the method to three dimensions. We applied a numerically stable method for least squares ellipsoid fitting based on the ellipsoid fitting method (Li and Griffiths 2004) and enhanced direct ellipsoid fitting method (Maini 2006) to point data sampled with the Rayburst sampling algorithm.

The ellipsoid surface can be described by a quadric equation as follows:

3 $$ {ax}^2+{by}^2+{cz}^2+ dxy+ exz+ fyz+ px+ qy+ rz+k=0 $$

The parameter vectors can be defined as follows:

4 $$ \boldsymbol{a}={\left[a\ b\ c\ d\ e\ f\ p\ q\ r\ k\right]}^T,\kern0.36em \boldsymbol{x}=\left[\ {x}^2\kern0.5em {y}^2\ {z}^2\  xy\  xz\  yz\ x\ y\ z\ 1\right] $$

The parameter ***a*** can be solved by sampling data with the ellipsoid fitting method.

First, sampled data are scaled (Maini 2006) for numerical stability:

5 $$ \hat{x}=\frac{x-{x}_{min}}{S_x},\hat{y}=\frac{y-{y}_{min}}{S_y},\hat{z}=\frac{z-{z}_{min}}{S_z} $$

where *x*_*min*_ , *y*_*min*_ , *z*_*min*_ are the minimum values of coordinates on the *X*, *Y*, and *Z* axes, respectively. *S*_*x*_ , *S*_*y*_ , *S*_*z*_ are scale factors for coordinate data on these three axes.

According to the ellipsoid fitting method (Li and Griffiths 2004), the main objective is to solve a generalized eigenvalue problem. ***a***_1_ is an eigenvector corresponding to the only positive eigenvalue of the eigenvalue system:

6 $$ \mathbf{M}{\boldsymbol{a}}_1=\lambda {\boldsymbol{a}}_1 $$

Here, ***a***_1_ is part of the parameter vector ***a*** **,**

7 $$ \boldsymbol{a}=\left({a}_1^T,{a}_2^T\right),{\boldsymbol{a}}_1={\left(a\ b\ c\ d\ e\ f\right)}^T,{\boldsymbol{a}}_2={\left(g\ h\ k\ l\right)}^T $$

*M* is a matrix for the sampled data and a constraint condition of the ellipsoid equation that is defined as follows:

8 $$ \mathbf{M}={\boldsymbol{C}}_1^{-1}\left({\boldsymbol{S}}_1-{\boldsymbol{S}}_2{\boldsymbol{S}}_3^{-1}{\boldsymbol{S}}_2^T\right) $$

In Eq. (8), ***S*** is a scatter matrix that can be calculated from the sampled data *p*_*i*_(*x*_*i*_, *y*_*i*_, *z*_*i*_):

9 $$ {\displaystyle \begin{array}{l}\mathbf{S}={\boldsymbol{D}}^{\boldsymbol{T}}\boldsymbol{D},\boldsymbol{D}={\left({\boldsymbol{X}}_1\ {\boldsymbol{X}}_2\dots {\boldsymbol{X}}_{\boldsymbol{n}}\right)}^T,\\ {}{\boldsymbol{X}}_{\boldsymbol{i}}={\left({x}_i^2\ {y}_i^2\ {z}_i^2\ {x}_i{y}_i\ {x}_i{z}_i\ {y}_i{z}_i\ {x}_i\ {y}_i\kern0.5em {z}_i\ 1\right)}^T\end{array}} $$

10 $$ \mathbf{S}=\left(\begin{array}{cc}\hfill {\boldsymbol{S}}_1\hfill & \hfill {\boldsymbol{S}}_2\hfill \\ {}\hfill {\boldsymbol{S}}_2^T\hfill & \hfill {\boldsymbol{S}}_3\hfill \end{array}\right),\left\{\begin{array}{c}\hfill {\boldsymbol{S}}_1={\boldsymbol{D}}_1^T{\boldsymbol{D}}_1\hfill \\ {}\hfill {\boldsymbol{S}}_2={\boldsymbol{D}}_1^T{\boldsymbol{D}}_2\hfill \\ {}\hfill {\boldsymbol{S}}_3={\boldsymbol{D}}_2^T{\boldsymbol{D}}_2\hfill \end{array}\right. $$

***S***_1_ , ***S***_2_ , ***S***_3_ are partitioned matrices of the matrix ***S*** with dimensions of 6 × 6, 6 × 4, and 4 × 4, respectively (Li and Griffiths 2004; Halir 1999).

The matrix ***C*** provides a constraint for ellipsoid fitting and was set to 4*J* − *I* ^2^ = 1 in this study, based on Li and Griffiths’ work. According to the equation given by Li and Griffiths, the matrix ***C*** can be calculated as.

$$ \boldsymbol{C}=\left(\begin{array}{cc}\hfill {\boldsymbol{C}}_1\hfill & \hfill {0}_{6\times 4}\hfill \\ {}\hfill {0}_{4\times 6}\hfill & \hfill {0}_{4\times 4}\hfill \end{array}\right) $$, where

11 $$ {\boldsymbol{C}}_1=\left(\begin{array}{c}\hfill \begin{array}{ccc}\hfill -1\hfill & \hfill 1\hfill & \hfill 1\hfill \\ {}\hfill 1\hfill & \hfill -1\hfill & \hfill 1\hfill \\ {}\hfill 1\hfill & \hfill 1\hfill & \hfill -1\hfill \end{array}\ \hfill \\ {}\hfill\ \begin{array}{ccc}\hfill -1\hfill & \hfill\ \hfill & \hfill\ \hfill \\ {}\hfill\ \hfill & \hfill -1\hfill & \hfill\ \hfill \\ {}\hfill\ \hfill & \hfill\ \hfill & \hfill -1\hfill \end{array}\hfill \end{array}\right) $$

***a***_1_ is the eigenvector associated with the only positive eigenvalue of the eigenvalue system in Equation (6). ***a***_2_ is calculated by

12 $$ {\boldsymbol{a}}_2=-{\boldsymbol{S}}_3^{-1}{\boldsymbol{S}}_2{\boldsymbol{a}}_1 $$

The parameters of the ellipsoid equation are calculated through this procedure. Then, the soma segmentation results are generated with the ellipsoid equation. Finally, a volume threshold is set according to the actual soma size to filter the correct soma regions.

## Results

The proposed method was validated on a workstation (Intel Corel i7-4810MQ 2.8 GHz, 16 GB RAM, NVIDIA Quadro K3100 M, Microsoft Windows 7). The test data contained four image stacks: three were from the fMOST system labeled with green fluorescence (Gong et al. 2013) and the last was from VANO (Peng et al. 2009). The performance of the proposed method was evaluated in terms of soma localization or segmentation for the datasets. Table 1 presents the main parameters of our proposed method.

**Table 1:** Parameter selection in experiments

Parameter	Value	Notes
Sigma of LoG filters	1–4	A multi-scale LoG filter can enhance the soma blob at different sizes. A larger sigma consumes more time, so proper sigma selection is important.
Volume threshold	fMOST data: 4200 (voxel) VANO data: 115 (voxel)	Eliminates the small regions of the binary image. The value is set based on the soma size.
Jitter height	0–2	Eliminates the distance map jitter of height *h*. The value selection depends on the real soma radius.
Rays	258 rays	Controls the sampling and speed precision of the Rayburst sampling algorithm.
*Rc*	fMOST data: 14 (voxel) VANO data: 6 (voxel)	*Rc* is the mean soma radius used to estimate the soma localization.

### Evaluation of Soma Localization

The recall and precision were used to evaluate the soma localization results. The ground truth for the evaluation was determined manually. We did not consider all broken somata for both the ground truth and results. A soma was accepted if the Euclidean distance between the automatically located soma centroid and manually labeled soma centroid was less than *Rc*, which is the mean soma size described by the radius of somata in images. About 30 somata were extracted randomly from the image stacks. The maximum length of their axes were used to calculate Rc, which was set to 7 μm for the fMOST datasets and 1.5 μm for the VANO dataset.

The recall and precision were defined as follows:

13 $$ \mathrm{recall}=\frac{\mathrm{N}\left(\mathrm{correctly}\right)}{\mathrm{N}\left(\mathrm{all}\right)} $$

14 $$ \mathrm{precision}=\frac{\mathrm{N}\left(\mathrm{correctly}\right)}{\mathrm{N}\left(\mathrm{groundtruth}\right)} $$

where N(correctly) denotes the number of somata correctly located by the automatic algorithm and N(groundtruth) is the soma number of the ground truth. N(all) represents the number of somata located by the automatic algorithm.

**Fig. 6:**
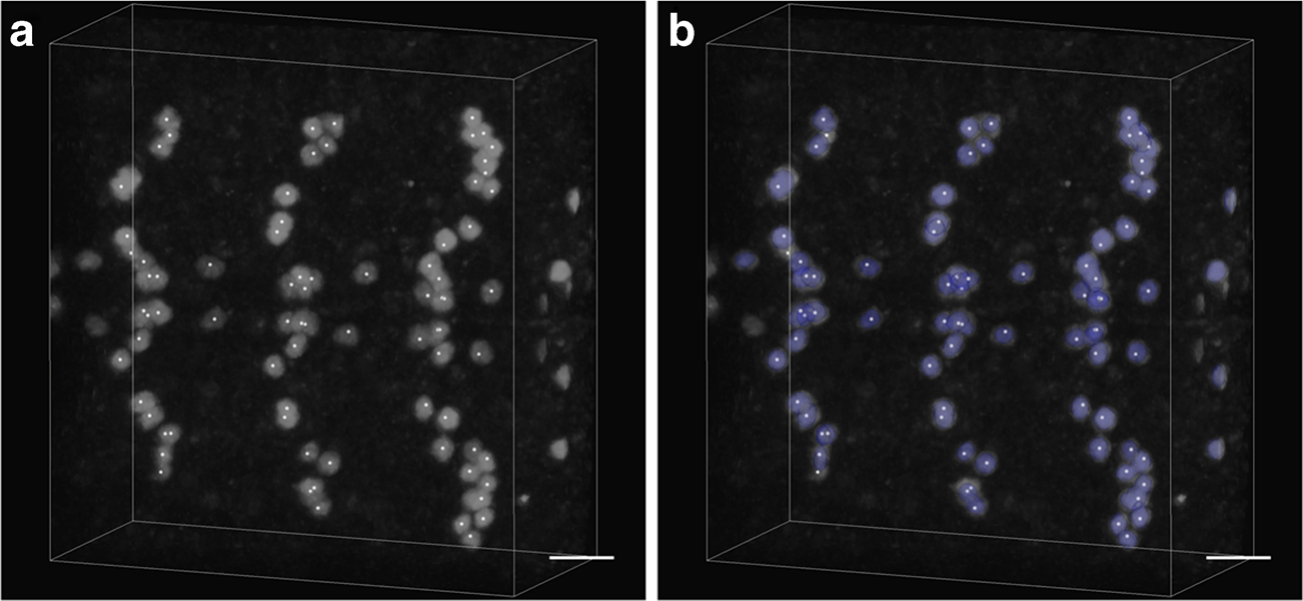
Segmentation results for the VANO dataset: (a) original image stack and (b) result of proposed method. The segmentation results are in blue, and the soma centroid is in white. The scale bar is 10 μm

The image stack from VANO contained 80 cells and had dimensions of 236 × 249 × 105 voxels. Cells were clustered in this image stack, as shown in Fig. 6(a). As shown in Fig. 6(b), most of the cells in the image stack were segmented with the proposed method. The proposed method also showed good results for touching somata. The soma segmentation had a runtime of 4.8 s with a recall of 96.2% and precision of 95.0%.

The datasets from the fMOST system included neuronal somata and neurite. All of these image stacks contained touching somata. Table 2 presents the results and soma segmentation runtime,(code was implemented in MATLAB). The precision and recall were more than 90%, excluding the broken somata around the image border. The proposed method was able to segment most of the somata in the image stack. The proposed method was compared with the original Rayburst sampling algorithm (Rodriguez et al. 2006), whose results are also presented in Table 2. The original Rayburst sampling was added to our pipeline for soma segmentation to generate results.

**Table 2:** Results of soma localization

Image Stack	Size (μm^3^)	Total somata	Rodriguez’s method		Proposed method		Soma segmentation runtime
			Recall	Precision	Recall	Precision	
**1**	175 × 175 × 62.5	27	100.0%	85.2%	96.3%	96.3%	39.0 s
**2**	225 × 225 × 75	53	91.5%	81.1%	96.2%	96.2%	31.7 s
**3**	225 × 225 × 75	33	96.8%	93.9%	100.0%	97.0%	27.7 s

Image stacks 1 and 2 contained more touching somata than image stack 3 (7 complete touching somata in image 1, 12 complete touching somata in image 2, 2 complete touching somata in image 3). The proposed method clearly performed better than original Rayburst Sampling Algorithm. The original Rayburst sampling algorithm could not process touching somata well and missed touching soma pairs. The original Rayburst sampling tended to generate bigger soma segmentation results than the proposed method.

For the proposed method, the main error was from flat or elongated somata. Elongated somata can result in more than one position and be segmented as more than one soma. The centroid of flat soma with a small distance value can be missed in the jitter elimination step, these types of somata can cause the regional maximum region (see C in Fig. 3) to be missed when they are touching other large somata.

### Evaluation of Soma Segmentation

The segmentation results were also evaluated by using the overlap ratio. This describes the ratio of the overlap parts for two regions and is defined as follows:

15 $$ \mathrm{overlap}\  \mathrm{ratio}=\frac{\mathrm{Op}}{\left(\mathrm{Seg}+\mathrm{GT}\right)/2} $$

16 $$ \mathrm{Op}=\mathrm{Seg}\cap \mathrm{GT} $$

where, o_p_ is the size of the overlapping region of the segmentation result and ground truth for one soma, Seg is the segmentation region of one soma, and GT is the region size of the manually determined ground truth.

A small image stack (145 μm × 145 μm × 62.5 μm) containing 30 somata was extracted from image stack 1 for evaluation, and 23 somata with clear and complete boundaries in this image stack were reconstructed manually.

The original Rayburst sampling algorithm (Rodriguez et al. 2006) and improved Rayburst sampling algorithm for soma segmentation were also compared. To generate the segmentation results, the original Rayburst sampling algorithm was combined with the ellipsoid fitting method. The soma centroids for the original Rayburst sampling algorithm were labeled manually. The intensity threshold was set to 146 based on the manual trials.

**Fig. 7:**
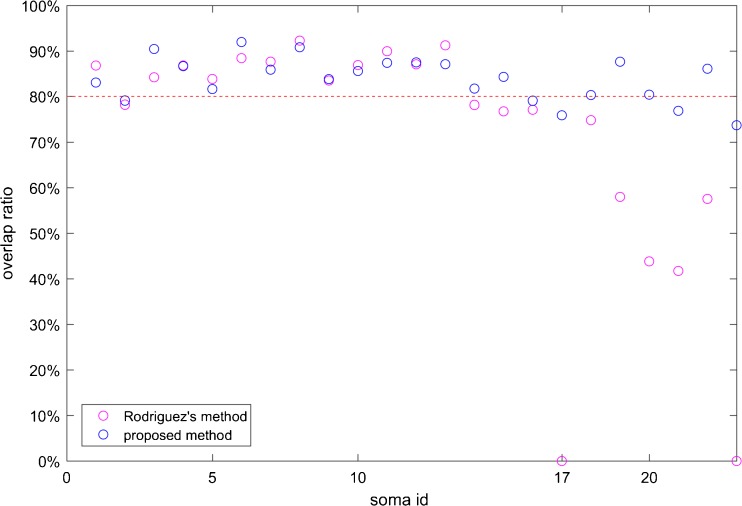
Overlap ratio of segmentation results: overlap ratio of 23 somata in the image stack from image 1. Touching somata have IDs of 17–23; the others are isolated somata

**Fig. 8:**
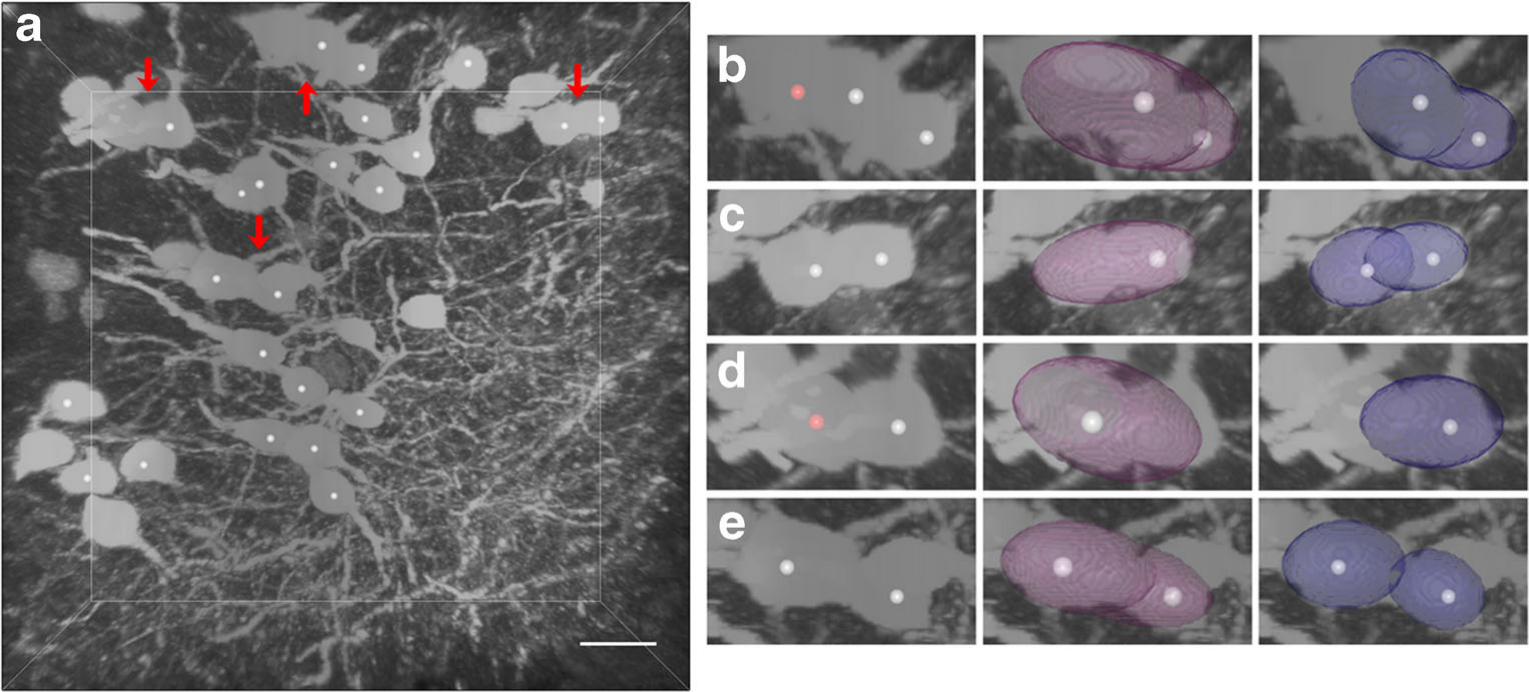
Segmentation results for the touching somata. (a) Original image stack. Touching somata are labeled with arrows. The segmentation results of the touching somata are shown in (b)–(e), where the results of the proposed method and original Rayburst sampling algorithm are in blue and purple, respectively. The complete soma are labeled by white balls, the broken soma are labeled by red balls. The scale bar is 15 μm

Fig. 7 shows the overlap ratio of somata in this image stack. The performances of these two methods for isolated somata were similar: the mean overlap ratio was 83.44% for the proposed method and 84.35% for the original Rayburst sampling algorithm. The main difference came from touching somata with IDs of 17–23; see Figs. 8(b)–(e).

**Fig. 9:**
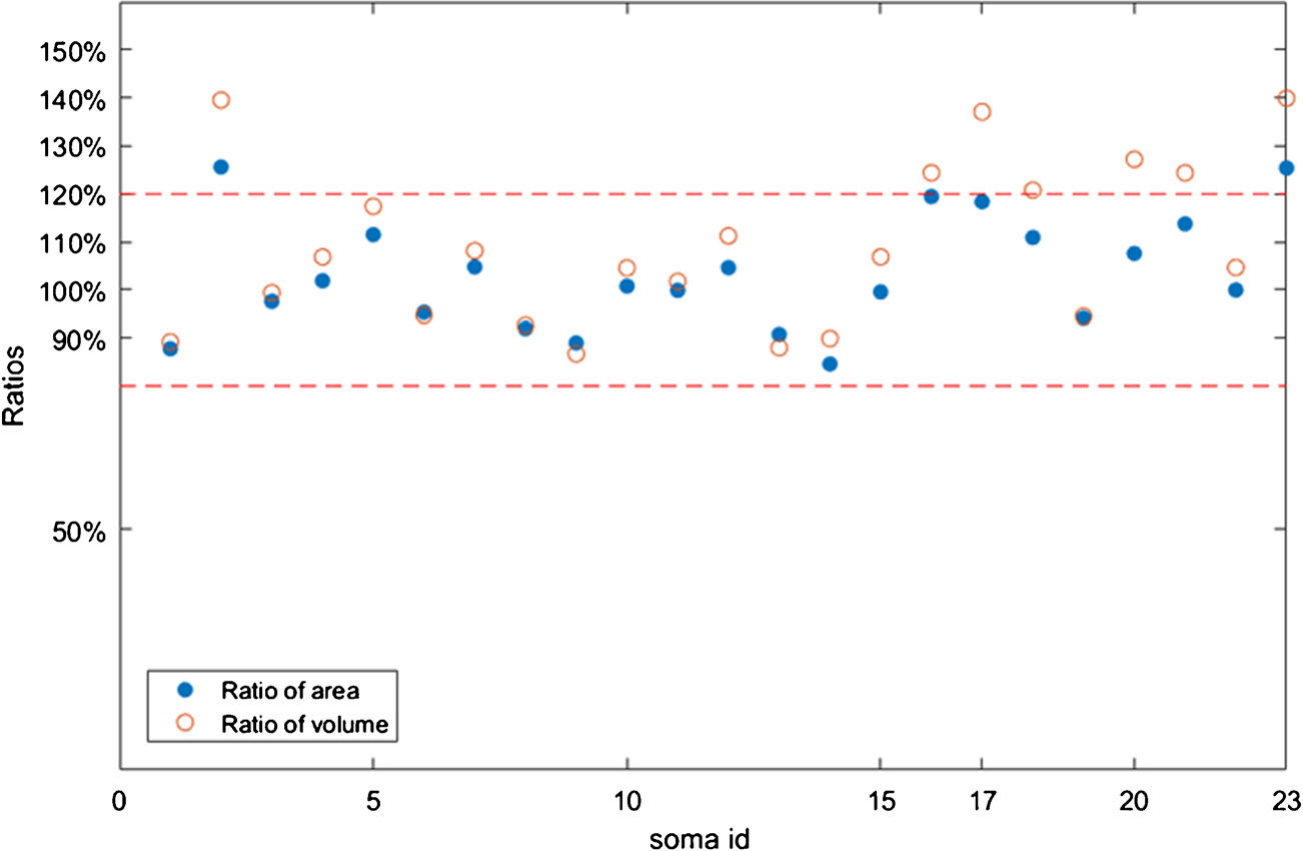
Ratios of segmentation results

**Fig. 10:**
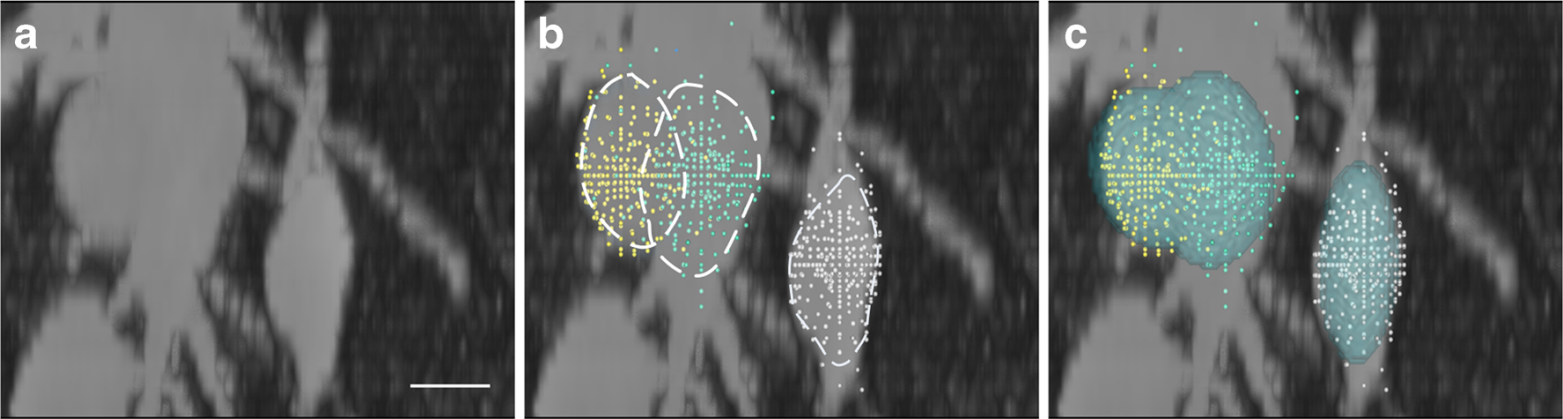
Sampling points on the soma surface: (a) Maximum intensity projection (MIP) of the original image stack. (b) Actual sampling points on soma surface overlaid in an image stack. The two touching somata are in green and yellow, the isolated one is in white, and the dashed line represents the rough shape of the region surrounded by the sampling points. (c) Segmentation results are visualized by transparent green overlaying the sampling points. The scale bar is 10 μm

The touching somata are labeled by arrows in Fig. 8(a). The results for touching somata are shown in Figs. 8(b)–(e). The proposed method performed better for touching somata. The results indicated that a touching soma pair with little overlap could be accurately determined.

In Fig. 7, the soma with an ID of 17 and 23 was segmented with a low overlap ratio. This is the left soma in Fig. 8(c) and the complete soma in Fig. 8(d). These two somata had a large touching region. In general, each soma matched a larger segmentation result than itself. The main conclusion could be that the touching region caused sampling rays to go through the best stopping position, which influenced the modeling results. This problem was obvious in the results with 66 sampling rays.

In terms of the neuron quantitative analysis, soma size characteristics such as the volume and surface area are important indices. Therefore, we compared these basic soma properties for evaluation. The ratios of the ground truth to the automatic segmentation result were used for the evaluation:

17 $$ \mathrm{volume}\  \mathrm{ratio}=\frac{\mathrm{Seg}\left(\mathrm{volume}\right)}{\mathrm{GT}\left(\mathrm{volume}\right)} $$

18 $$ \mathrm{area}\  \mathrm{ratio}=\frac{\mathrm{Seg}\left(\mathrm{area}\right)}{\mathrm{GT}\left(\mathrm{area}\right)} $$

where Seg(volume) is the volume of the segmentation result, GT(volume) is the volume of the ground truth, Seg(area) is the area of the segmentation result, and GT(area) is the area of the ground truth.

The proposed method provided preferable results in terms of the soma size. The ratios of most segmentation results were in the range of 100% ± 20%, which included touching somata (Fig. 9). The generated ellipsoid models described the soma size relatively accurately.

Above all, the worst segmentation results were when two somata had a large touching region. For example, for the two somata in Fig. 8(c), one soma had low overlap ratio (ID 23 in Fig. 7). This may be because the touching region caused more sampling rays to go through the best stopping position and generated a larger result than itself. Moreover, one soma can be missed if touching somata are so close that there is only one regional maximum region in these somata; these touching somata could look like a single soma.

Fig. 10 visualizes the sampling and segmentation results for our method. For the touching somata, most of the sampling points from one soma centroid were clustered around the matching soma surface, and several false sampling rays terminated in the other soma, as shown in Fig. 10(b). For isolated sampling points, some were slightly far from the best position. Modeling the surface by using the ellipsoid fitting results reduced the influence of false sampling points, as shown in Fig. 10(c). The model contours excluded the bad sampling points approximated by the manually and roughly labeled soma contours, as shown in Fig. 10(b).

## Discussion

Our algorithm mainly comprises the Rayburst sampling algorithm and ellipsoid fitting. These two parts contain simple algorithms that were performed with high efficiency, as indicated in Table 2. However, the preprocessing step occupied more than 50% of the total runtime because the multi-LoG filters for the 3D image dataset are too slow. Parallelizing the technique could be a solution to improving the speed of the preprocessing and Rayburst sampling algorithm (Yan et al. 2013).

The DT-based locating method found most of the somata in images in practice, but it generated many false locations in thick neurites or noise blob structures. Thus, we refined the initial result by using H-dome transform and soma size information. H-dome transform can delete redundant local maximum value points in one soma. The results showed that most somata in an image stack could be located. H-dome transform can delete many false locations in a short time, and the soma volume threshold can be set according to the minimum soma size to filter the correct somata. During the segmentation flow, candidate soma centroids that overlap in the generated segmentation would be deleted for efficiency.

On the other hand, the stopping condition for ray casting provided a better performance with the proposed method than with the original Rayburst sampling algorithm. The stopping condition for the original Rayburst sampling is based on voxel intensity, which could not give the best stopping position for the casting ray. For the variant of the original Rayburst sampling algorithm using the image gradient to rectify the sampling results, the results showed that somata close to each other could not be processed well (Yan et al. 2013).

Consequently, the proposed method could make most of the rays stop around the correct location. However, there were still some incorrect rays in the touching somata. One reason for this is that the EDT for the irregular structure may have generated soma regional minima inside. Somata with a large touching region could be another reason in that they make many rays go through the best location. One solution could be using DT with intensity information (Xiao and Peng 2013).

To avoid a rough surface, the ellipsoid model was chosen to fit the soma shape (Jung and Kim 2010) instead of a triangle mesh. The results showed a relatively high overlap ratio between the model and gold standard. Therefore, this ellipsoid model can describe the soma morphology relatively accurately.

As discussed in the previous section, most of the errors arose from irregularly shaped somata. Another main reason is the limitations of the ellipsoid because it is unable to describe some kinds of irregular shapes, even though the surface sampling was sufficiently accurate. Increasing the sampling rays could make the generated ellipsoid model approximate the soma shape.

In the fMOST data, many somata were shaped like ellipsoids or spheres, so the ellipsoid fitting method could be efficient. For different datasets, our method could produce better results after the preprocessing step is changed. In terms of soma morphology, our model can approximate most somata with a relatively high overlap ratio, as shown in Fig. 7. Under less packed conditions, this model could be used to obtain rough segment results.

## Conclusion

This paper proposes an automatic soma segmentation method suitable for datasets with touching soma distributions. The proposed method contains three main parts: (1) soma detection based on DT and local maximum searching and refining; (2) an improved Rayburst sampling algorithm for isolated and touching soma surface detection; and (3) ellipsoid fitting to generate the segmentation results.

The results of experiments on fMOST and VANO datasets showed that the proposed method can perform with relatively high accuracy on datasets contain touching somata. In terms of soma quantification, the soma size is an important factor (Uylings and Van 2009). An evaluation of the soma sizes showed that the volume and surface area of the segmentation results had relatively high accuracy, which indicates that our method can be used for efficient soma quantification analysis.

The parameterized model generated with our method exhibited a relative high overlap ratio in the experiments. The results from this model can be used as a rough geometric model for qualitatively analysis of soma morphology or provide a basis for neuronal electrophysiology reconstruction and simulation with large-scale neural datasets.

## Information Sharing Statement

The source code is available at github: https://github.com/keepersecond/Soma-Segmentation.

## Electronic supplementary material

ESM 1 (DOCX 775 kb)

ESM 2 (DOCX 148 kb)
