# Supplementary figures and images for: Touching Soma Segmentation Based on the Rayburst Sampling Algorithm
Source: Neuroinformatics. 2017 Sep 22;15(4):383–93. doi: 10.1007/s12021-017-9336-y (PMC5671566; doi:10.1007/s12021-017-9336-y)

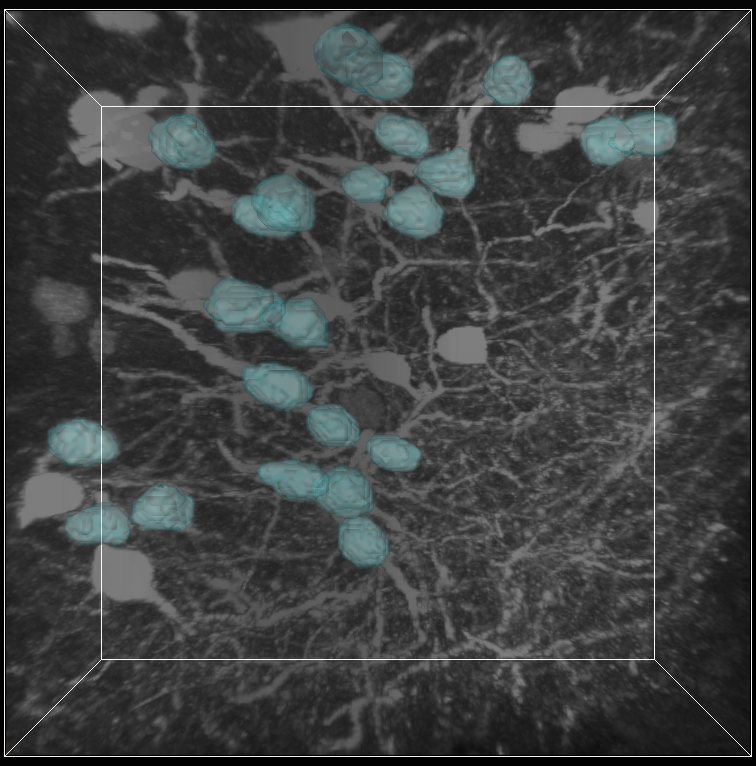


**Fig.1** Ground Truth of image stack

Supplement: Supplementary file 1 — (DOCX 775 kb) [file 12021_2017_9336_MOESM1_ESM.docx]
