# Supplementary material for: Touching Soma Segmentation Based on the Rayburst Sampling Algorithm
Source: Neuroinformatics. 2017 Sep 22;15(4):383–93. doi: 10.1007/s12021-017-9336-y (PMC5671566; doi:10.1007/s12021-017-9336-y)
Supplement: Supplementary file 2 — (DOCX 148 kb) [file 12021_2017_9336_MOESM2_ESM.docx]

Ratios of Original Rayburst Sampling Algorithm


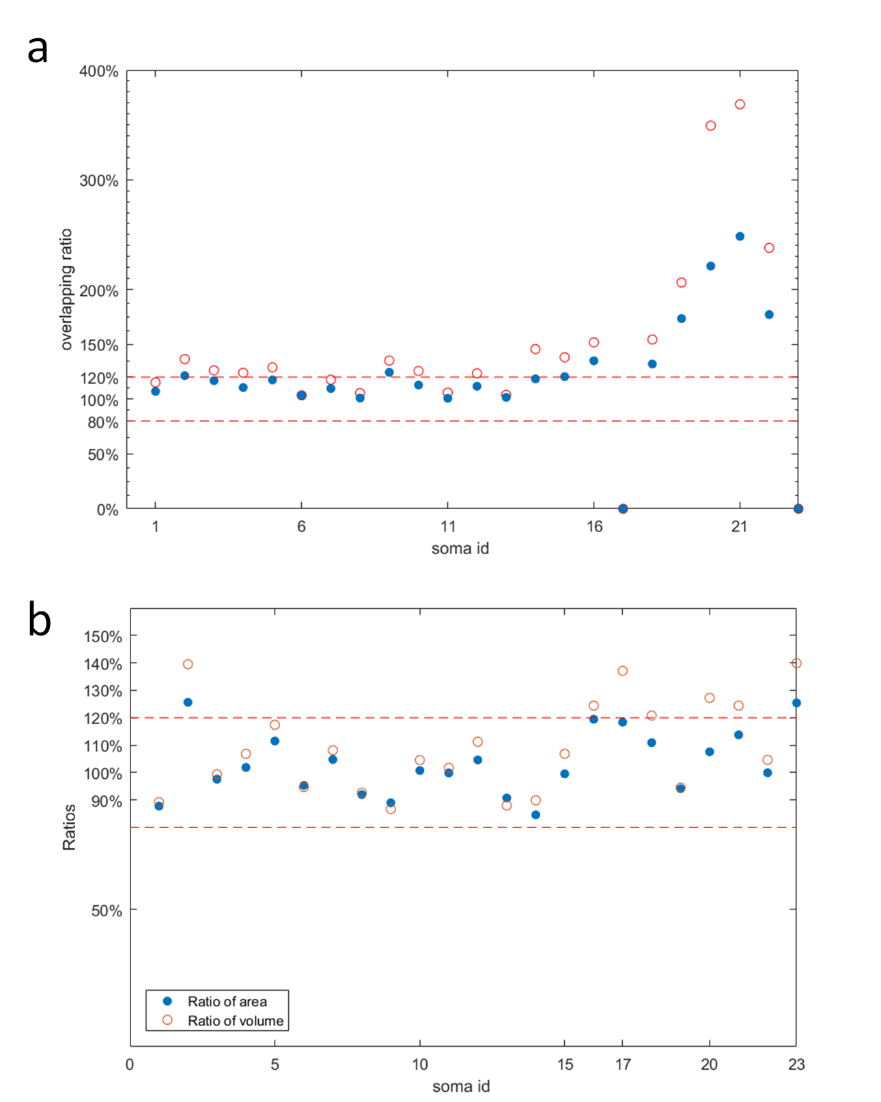


**Fig.2** Ratios of segmenation: (a) Ratios of results from original Rayburst sampling algorithm,(b) Ratios of results from proposed method. Touching somata have IDs of 17 to 23, the others are isolated somata.

For original Rayburst Sampling Algorithm, mean value of volume ratio is 143.6%, mean value of area ratio is 120.2%. For proposed method, mean value of volume ratio is 109.1%, mean value of area ratio is 103.3%. In Fig.2, touching somata with id from 17 to 23, mean difference comes from touching somata.
